# Hospital emergency department utilisation rates among the immigrant population in Barcelona, Spain

**DOI:** 10.1186/1472-6963-8-51

**Published:** 2008-03-03

**Authors:** Andrea Buron, Francesc Cots, Oscar Garcia, Oriol Vall, Xavier Castells

**Affiliations:** 1 Health Services Evaluation and Clinical Epidemiology Department, IMIM-Hospital del Mar, Barcelona, Spain; 2 Educational Unit of Preventive Medicine and Public Health IMAS-UPF-ASPB, Barcelona, Spain; 3 Neuropsychopharmacology Programme, Childhood and Environment Research Unit, Paediatric Unit, IMIM-Hospital del Mar, Barcelona, Spain; 4 Departament de Pediatria, Obstetrícia i Ginecologia, i Medicina Preventiva, Universitat Autònoma de Barcelona, Barcelona, Spain

## Abstract

**Background:**

The recent increase in the number of immigrants of Barcelona represents a challenge for the public healthcare system, the emergency department being the most used healthcare service by this group. However, utilisation rates in our environment have not yet been studied. We aimed to compare emergency department utilisation rates between Spanish-born and foreign-born residents in a public hospital of Barcelona.

**Methods:**

The *s*tudy population included all adults residing in the area of study and visiting the emergency department of Hospital del Mar in 2004. The emergency care episodes were selected from the Emergency Department register, and the population figures from the Statistics Department of Barcelona. Emergency care episodes were classified into five large clinical categories. Adjusted rate ratios (RR) of utilisation among foreign-born vs. Spanish-born residents were assessed through negative binomial regression.

**Results:**

The overall utilisation rate was 382 emergency contacts per 1,000 persons-years. The RR for foreign-born versus Spanish-born residents was 0.62 (95% CI: 0.52; 0.74%). The RR was also significantly below one in surgery (0.51, 95% CI: 0.42; 0.63), traumatology (0.47, 95% CI: 0.38; 0.59), medicine (0.48, 95% CI: 0.38; 0.59) and psychiatry (0.42, 95% CI: 0.18; 0.97). No differences were found in utilisation of gynaecology and minor emergency services.

**Conclusion:**

The overall lower utilisation rates obtained for foreign-born residents is consistent with previous studies and is probably due to the "healthy immigrant effect". Thus, the population increase due to immigration does not translate directly into a corresponding increase in the number of emergency contacts. The lack of differences in minor and gynaecological emergency care supports the hypothesis that immigrants overcome certain barriers by using the emergency department to access to health services. The issue of healthcare barriers should therefore be addressed, especially among immigrants.

## Background

Spain has traditionally been a country of emigration, with immigration being a relatively recent phenomenon. According to the Statistical Office of the European Community, in 2004, Spain was the European country that received the highest number of immigrants in absolute terms, with 652,000 immigrants, representing most of the 1.7% total population growth [1]. The Spanish immigrant population is mainly economic, i.e. immigrants are from countries that are economically disadvantaged compared with Spain, and by far the most frequent countries of origin (and their percentage over the total immigrant population in Spain in January 1^st^, 2005) are Morocco (12.7%), Ecuador (11.1%), Rumania (7.1%) and Colombia (6.6%) [2]. Unlike other European countries such as the Netherlands, the percentages of refugees fleeing from political and ethnic persecution or civil war are very low.

Catalonia is one of the Autonomous Communities of Spain attracting the greatest number of immigrants [2], most of whom settle in Barcelona. In the last 5 years, immigrants have formed an increasing part of the population in Barcelona, their number rising from 74,019 in January 2001 to 260,058 in January 2006 [3]. The percentage of the immigrant population rose during the same period from 4.9% to 15.9%, figures similar to those found in countries with a longer tradition of immigration such as the United Kingdom, Germany, Austria, and the Netherlands [4]. Immigrants living in Barcelona tend to gather in certain areas of the city, Ciutat Vella being the district with the highest percentage of immigrants (38.5% in 2006). The most frequent nationalities among immigrants in the district regard to Pakistan (15.9% of all immigrants in Ciutat Vella come from this country), Morocco (11.5%), Philippines (10.9%) and Ecuador (7.5%) [3].

One of the most worrisome political issues regarding immigration is the ability of healthcare services to serve the increasing number of immigrants, and to provide equity of access to healthcare services among this population [5]. The Spanish National Health System provides universal access to healthcare; this access is free at the point of use to all residents and user co-payments are restricted to pharmaceutical and dental care. The health card, issued by the Health Department of each Autonomous Community through the primary care centers, is the official document that ensures this health coverage and is the basis of the health register. In Spain, the right of the immigrant population to health and healthcare is regulated by the set of laws dealing with immigration and naturalisation [6]: healthcare is guaranteed for minors and pregnant women, persons with a medical emergency, and immigrants registered with their local census bureau. Therefore, for all immigrants living in Spain, the principles of public, free and universal services mainly apply to the emergency care services, whereas access to primary and specialized care might present several barriers. These barriers include the need to have a health card, lack of knowledge about how to access these services, and difficulties in making an appointment (language, time schedules, waiting lists, etc.). To obtain a health card, the only requisite is proof of place of residence through the certificate of registration. In the last few years, local policy has eased the process of registration among immigrants and thus the percentage of the unregistered foreign-born population, although unknown, is believed to be very small.

Economic indicators of poverty have been related to poorer health and higher healthcare service utilisation [7,8]. Given that most of the immigration in the study area comes from low-income countries and is therefore economic [3], the impact of the increasing immigrant population on the Catalan healthcare system is thought to be high and providing equity of access to healthcare services represents a challenge.

The few studies describing healthcare services utilisation by immigrants in Spain tend to highlight their overall under-utilisation of services compared with non-immigrants, the preponderant use of public services over the private sector, and the greater use of emergency rooms than of other services to counteract access barriers to other health services [5,9-12]. To our knowledge, no previous studies have taken the reference population into account and have therefore not been able to compare healthcare utilisation in relative terms.

We decided to compare utilisation rates of emergency services for several reasons. Spanish legislation ensures emergency room utilisation to everyone, regardless of origin, nationality or legal status [6]. In our catchment area, immigrants represent a much higher percentage of emergency visits than any other hospital visits [9]. Thus, the purpose of this study was to assess hospital emergency department utilisation among registered Spanish-born and foreign-born adults in Barcelona in 2004. Because of the access barriers to other healthcare services that immigrants may encounter and their poor socioeconomic position [12-17], our hypothesis was that the emergency department utilisation rate would be higher among immigrants than among the host population.

## Methods

Many different definitions of immigrants have been used in the literature and the most appropriate way to register and study immigrant issues is still being debated [18]. The present study defines immigrant status by country of birth, and therefore the population will be divided into Spanish-born and foreign-born. We chose to dichotomise the origin of the population to simplify the analysis and reporting of results, and because previous research in our country [9,19], as well as from other countries [20-22], showed no differences among distinct groups of immigrants in healthcare utilisation.

A cross-sectional study was designed to evaluate the utilisation rates of registered Spanish-born and foreign-born adults visiting the emergency department of a public teaching hospital, Hospital del Mar, in Barcelona in 2004. The study area comprises the four health districts of the hospital's catchment area in Ciutat Vella, which have the highest attraction index (this index is defined as the percentage of emergency contacts attended in our hospital out of all emergency contacts made by people in our hospital's catchment area and attended in all public hospitals of the area). In this area, the emergency department of Hospital del Mar is the only emergency service available for the population and therefore attends almost all the emergency visits.

The study population included all adults residing in the study area and visiting the emergency department of Hospital del Mar in 2004. Emergency care episodes were classified into five large clinical categories: obstetric and gynaecological, surgical, traumatological, medical, psychiatric, and minor emergencies. The information source for the care provided was the emergency department's patient register. Among other variables, sex, age, address, country of birth, and attending specialty were registered.

Calculation of the utilisation rate requires figures on the reference population. As population information was not available for unregistered people, we had to exclude emergency visits made by unregistered persons, whether belonging to the autochthonous or immigrant populations. We therefore excluded emergency department contacts made by persons without a health card, as registration is required to obtain one. The population aged less than 15 years was also excluded due to the difficulty of classifying their immigration status (the utilisation rate in this age group was determined mostly by their parents, and the country of birth of both parents was not always available; even when available, classification when one parent was foreign-born and the other Spanish-born remained a dilemma).

The source for the reference population living in the study area was the municipal register, since the last census in Spain was conducted in 2001 and immigrants in Spain constitute a rapidly changing population group [2]. The municipal registry is an administrative registry that includes all citizens in the municipality. Registration provides proof of current residence in the town for each resident and everyone living in Spain is required to register in the registry of the town of residence. On January 1^st ^of each year, these data are reviewed, analysed and published, both locally and nationally by the corresponding Statistics Departments. The reference population in our study included all registered citizens aged 15 years and older in the study area on January 1^st^, 2005, which was the best approximation available to the number of persons living in the study area in 2004. These data were obtained from the Statistical Department of Barcelona and were grouped by age, sex, place of birth, and area of residence.

The utilisation rate of a specific medical service, in a specific area and during a specific time period, is defined as the ratio of the total number of healthcare episodes performed in a specific service in individuals living in the area during that time period, divided by the total population living in the area. In this study, the outcome variable was the utilisation rate for each category of age, sex, and country of birth. The main explanatory variable was the patient's country of origin (Spain or elsewhere). Sex, age (15–49 years, 50–64 years, and 65 and older) and specialty (obstetric and gynaecological, surgical, traumatological, medical, psychiatric, and minor emergencies) were included in the model as covariates. The age categorisation was chosen to have a sufficient number of individuals when the database was aggregated by age, sex, and specialty.

To compare the distribution between Spanish-born and foreign-born emergency episodes by sex, age and emergency department specialty, a *chi-square *test was used. The units of analysis were determined by the categories of age, sex, and birth in Spain or elsewhere; information on emergency visits and the population were linked by groups, i.e., each unit therefore had a number of emergency episodes and a number of registered persons. Comparison of the crude utilisation ratios between Spanish-born and foreign-born individuals was performed through the utilisation rate ratio and its confidence intervals.

After crude rate ratios were calculated, a Poisson regression model was initially used to obtain an overall estimate of the effect of birth outside Spain on emergency department utilisation. Due to a high overdispersion value, a negative binomial regression model was then used, resulting in low overdispersion values. The negative binomial regression finally used had the following structure:

Log(Emergencies) = log(population) + β_1 _origin + β_2 _sex + β_3 _age + β_4 _specialty + error,

which was equivalent to

Utilisation rate = Exp (β_1 _origin + β_2 _sex + β_3 _age + β_4 _specialty + error)

All variables in the definitive model were significant except sex. However, we decided to retain this variable in the model because of its importance as an adjusting variable in epidemiological studies; furthermore, differences between males and females were statistically significant in crude utilisation ratios and in the surgery model. All possible interactions were tested but only the interaction between age and specialty was significant, which was therefore included.

A valid model for all healthcare episodes in all specialties was obtained; then, because specialty was significant in the general model and there was an interaction between age and specialty, a model for each specialty was developed to elucidate the effect of birth outside Spain on each specialty utilisation. The model for each specialty had the same structure, except for the variable of "specialty", which was omitted. In the model for Gynaecology and Obstetrics the variable of sex was also removed:

Utilisation rate _Sp _= Exp (β_1 _origin + β_2 _sex + β_3 _age + error);

Utilisation rate _Gyn/Obst _= Exp (β_1 _origin + β_3 _age + error),

in which "Sp" is any specialty except for Gynaecology and Obstetrics and "Gyn/Obst" is Gynaecology and Obstetrics.

The denominator of the utilisation rate was composed of groups of persons while the numerator consisted of emergency visits. Emergency visits were therefore not independent, since some persons visited the emergency department more than once in the study period. No differences were found in the distribution by age and sex of persons making more than one visit during this period and that in the total number of emergencies studied.

The data analysis for this study was generated using SPSS for Windows version 12.0 and SAS software, Version 9.1 [23,24].

## Results

**Figure 1:**
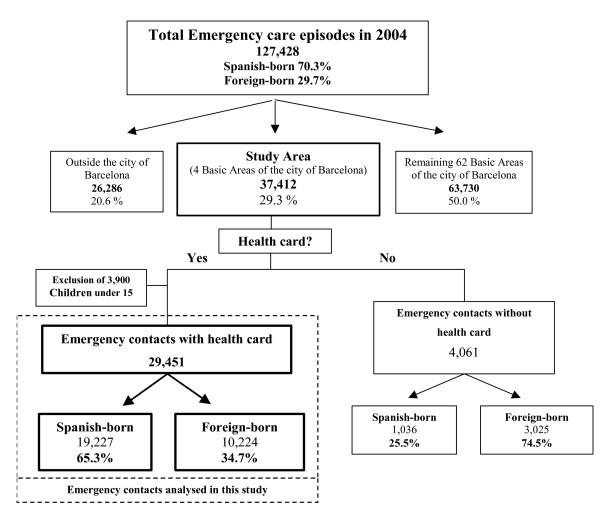
Flow chart of the emergency care episodes in Hospital del Mar in 2004 included in this study.

In 2004, there were 127,428 emergency visits to the emergency department of Hospital del Mar in Barcelona. Of these, 29.3% (37,412) were made by persons living in the study area. We excluded 21.0% of visits made by foreign-born residents and 4.5% of those made by Spanish-born residents due to lack of a health card. Visits made by children under 15 years old were also excluded. Of the 29,451 visits finally analysed, 34.7% were made by foreign-born residents (Figure 1).

More emergency contacts were made by women (53.7%) in the Spanish-born group than in the foreign-born group, in which most emergency visits (57.6%) were made by men (Table 1). Among foreign-born residents, approximately 90% of the emergency contacts were made by the group aged between 15 and 49 years old and only 2.7% were made by the group aged over 65 years; in contrast, in the Spanish-born group, there was a higher frequency of visits made by elderly (31.6%) and a lower frequency of visits made by the middle-aged (50.8%). In relative terms, foreign-born residents more frequently used the specialties of gynaecology and obstetrics and minor emergency care than did the Spanish-born residents.

**Table 1:** Distribution of Hospital del Mar's emergency contacts among Spanish-born and foreign-born residents living in the study area by sex, age and emergency specialty (N = 29,451).

	**Spanish-born**	**Foreign-born**	**Total**
**Total, n (%)**^a^	19,227 (65.3%)	10,224 (34.7%)	29,451 (100.0)
**SEX, n (%)**^a^			
**Male**	8,907 (46.3%)	5,887 (57.6%)	14,794 (50.2%)
**Female**	10,320 (53.7%)	4,337 (42.4%)	14,657 (49.8%)
**AGE, n (%)**^a^			
**15–49**	9,758 (50.8%)	9,215 (90.1%)	18,973 (64.4%)
**50–64**	3,391 (17.6%)	733 (7.2%)	4,124 (14.0%)
**≥ 65**	6,078 (31.6%)	276 (2.7%)	6,354 (21.6%)
**EMERGENCY DEPARTMENT SPECIALTY, n (%)**^**a**^			
**Obstetric and gynaecological**	1,538 (8.0%)	1,491 (14.6%)	3,029 (10.3%)
**Surgical**	2,205 (11.5%)	635 (6.2%)	2,840 (9.6%)
**Traumatological**	2,779 (14.5%)	807 (7.9%)	3,586 (12.2%)
**Psychiatric**	527 (2.7%)	99 (1.0%)	626 (2.1%)
**Medical**	6,112 (31.8%)	1,399 (13.7%)	7,511 (25.5%)
**Minor Emergencies**	6,066 (31.5%)	5,793 (56.7%)	11,859 (40.3%)

^a ^*p *< 0.001

On January 1st, 2005, there were 77,097 persons living in the study area (Table 2); of these, 42.2% were foreign-born residents. Almost 90% of these residents were aged between 15 and 49 years old compared with less than half of the Spanish-born population. In contrast, there was a higher percentage of elderly individuals among the Spanish-born group.

**Table 2:** Sex and age distribution among the Spanish-born and the foreign-born population living in the study area (N = 77,097).

	**Spanish-born**	**Foreign-born**	**Total**
**Total, n (%)**^**a**^	44,589 (57.8%)	32,508 (42,2%)	77,097 (100%)
**SEX, n (%)**^**a**^			
**Male**	21,163 (47.5%)	20,350 (62.6%)	41,513 (53.8%)
**Female**	23,426 (52.5%)	12,158 (37.4%)	35,584 (46.2%)
**AGE, n (%)**^**a**^			
**15–49**	21,339 (47.9%)	28,968 (89.1%)	50,307(65.3%)
**50–64**	8,723 (19.6%)	2,687 (8.3%)	11,410 (14.8%)
**≥ 65**	14,527 (32.6%)	853 (2.6%)	15,380 (19.9%)

^a^*p *< 0.001

The crude utilisation ratios as well as the crude rate ratios (cRR) of foreign-born *vs *Spanish-born residents are shown in Table 3. The overall utilisation rate for the entire population studied was 382 emergency contacts per 1,000 persons-years. The utilisation rate was lower in foreign-born than in Spanish-born residents, the cRR being 0.73 (95% CI: 0.71; 0.74). The cRR of foreign-born men compared with that of Spanish-born men was 0.68 (95% CI: 0.67; 0.71) and that of women was 0.81 (95% CI: 0.78; 0.83). In all age ranges, the cRR of foreign-born versus Spanish-born residents was less than 1.

**Table 3:** Crude utilisation rates of the emergency department for the total study sample, the Spanish-born population and the foreign-born population; and crude rate ratio for the foreign-born vs the Spanish-born population.

	**Crude utilisation rate per 1,000 persons year TOTAL**	**Crude utilisation rate per 1,000 persons year Spanish-born residents**	**Crude utilisation rate per 1,000 persons year foreign-born residents**	**Crude rate ratio foreign-born vs. Spanish-born residents**	**95% Confidence interval**
**TOTAL**	382	431	315	**0.729**	(0. 715; 0. 744)
**SEX**					
**Male**	356	421	289	**0.687**	(0.669; 0.706)
**Female**	412	441	357	**0.810**	(0.78; 0.833)
**AGE**					
**15–49**	377	457	318	**0.696**	(0.680; 0.711)
**50–64**	361	389	273	**0.702**	(0.656; 0.750)
**≥ 65**	413	418	324	**0.773**	(0.701; 0.854)

The negative binomial regression model for all the emergency contacts analysed and after adjustment by age, sex and emergency specialty revealed an adjusted rate ratio (RR) for foreign-born versus Spanish-born residents of 0.62 (95% CI: 0.52; 0.74%). Table 4 shows the RR of country of origin, sex, and age determined through the negative binomial models in all the specialties. The utilisation rate was significantly lower in foreign-born than in Spanish-born residents in the specialties of surgery (0.51, 95% CI: 0.42; 0.63), traumatology (0.47, 95% CI: 0.38; 0.59), medicine (0.48, 95% CI: 0.38; 0.59) and psychiatry (0.42, 95% CI: 0.18; 0.97). In gynaecology, utilisation seemed to be higher among foreign-born women, although this difference was not statistically significant (1.43, 95% CI: 0.86; 2.35). No differences were found between foreign-born and Spanish-born residents in utilisation of minor emergency care. Minor and surgical emergencies were more frequent in men, while no statistically significant differences were found between men and women in the remaining specialties. The specialties of surgery, traumatology and medicine showed a direct association with age – the greater the age category, the higher the utilisation rate.

**Table 4:** Emergency department utilisation rate ratio in the study area for each emergency department specialty, derived from the negative binomial regression.

	**Obstetric and Gynaecological**	**Surgical**	**Traumatological**	**Medical**	**Psychiatric**	**Minor Emergencies**
**ORIGIN**						
**Spanish-born**	1.00	1.00	1.00	1.00	1.00	1.00
**Foreign-born**	1.43 (0.86; 2.35)	0.51 (0.43; 0.62)^c^	0.47 (0.38; 0.59)^c^	0.48 (0.39; 0.59)^c^	0.42 (0.18; 0.97)^a^	1.03 (0.92; 1.16)
**SEX**						
**Male**	-	1.68 (1.42; 1.98) ^c^	0.86 (0.69; 1.07)	1.04 (0.84; 1.30)	0.65 (0.30; 1.40)	1.22 (1.09; 1.36)^c^
**Female**	1.00	1.00	1.00	1.00	1.00	1.00
**AGE**						
**15–49**	1.00	1.00	1.00	1.00	1.00	1.00
**50–64**	0.11 (0.06; 0.19)^c^	1.35 (1.12; 1.62)^b^	1.06 (0.87; 1.36)	1.4 (1.13; 1.89)^b^	0.93 (0.41; 2.12)	0.74 (0.65; 0.84)^c^
**≥ 65**	0.12 (0.06; 0.22)^c^	2.07 (1.71; 2.51)^c^	1.22 (0.92; 1.61)	2.34 (1.79; 3.05)^c^	0.50 (0.15; 1.64)	0.46 (0.40; 0.53)^c^

^a^*p *< 0.05 ^b ^*p *< 0.01 ^c ^*p *< 0.001

## Discussion

The emergency department utilisation rate among foreign-born residents, once adjusted by age and sex, was 38% lower than that among Spanish-born residents. Moreover, across all age groups, the crude utilisation rates were lower in foreign-born than in Spanish-born residents. This result is consistent with previous reports of healthcare utilisation by the immigrant population and was probably due to the healthy immigrant effect [16,25], according to which recently arrived immigrants have better health status than native-born residents because of a previous "natural selection procedure" in each country of origin. This fact has also been proven in our country: the percentages of good and excellent self-perceived health were higher, and chronic morbidity was 40% less frequent among economic immigrants than among the rest of the population living in the city of Madrid, after adjusting for age, sex and educational level [26]. To our knowledge, the only study that has analysed utilisation rates by taking into account the reference population reported differential rates by groups of country, with some groups below (Western and European countries), some about the same (other non-Western-born residents) and others above (Somalia, Turkey and ex-Yugoslavia) the national utilisation levels [13]. However, in agreement with most studies using other approaches and not adjusting by the reference population [5,27-32], our finding of lower emergency department utilisation by immigrants suggests overall lower healthcare use in this population. Although patterns of immigration may differ between the countries in which those studies were made, they all suggest that there is no higher utilisation because of the fact of being immigrant as it is sometimes supported by some sectors of the society.

Lower utilisation among immigrants was found in all specialties except minor emergency care and gynaecology. The lack of differences in utilisation in these episodes between foreign-born and Spanish-born residents supports the hypothesis that immigrants overcome certain barriers by using the emergency department to access health specialties in preference to other routes [5,13,29,32]. This hypothesis is further supported by the finding that, in the same hospital, foreign-born residents account for a much larger percentage of emergency visits (23%) than of admissions (13%) and outpatient visits (6%) [33]. Although not statistically significant, the higher utilisation rates for gynaecology among foreign-born women was probably related to their higher fertility rates [34] and lower use of primary care gynaecologists (public and private) and antenatal care programs.

Minor and surgical emergencies were more frequent in men, probably because a high volume of women's minor and surgical emergencies were dealt within the gynaecological specialty of the emergency department, while the few cases of urology were added to surgical emergencies.

Exclusion of persons not carrying a health card from the emergency contacts analysed is consistent with including only the registered population in the denominator, since theoretically the only requirement to obtain a health card in Spain is to be registered in the census. Exclusion of these individuals also prevented the selection bias that would have been produced by including people who can use emergency services only. The Spanish-born cases excluded were mainly people living in other Autonomous Communities who were in the study area as tourists, and thus did not have a local health card and a smaller number of people living in the area but unregistered. The cases excluded among the foreign-born population included people living in the study area but not registered (whether undocumented or illegal or not) and tourists. Given that local policy has facilitated registration among immigrants (to ease their access to the health system, among other reasons), the number of foreign-born residents living in the study area but not registered was probably small, and probably involved those who had arrived most recently to the city and had not had the time, knowledge or need to register. In addition, the hospital is in a highly touristic area, and among the emergency contacts excluded, there was a higher proportion of people from European and high-income countries than among emergency contacts made by persons with a health card. Hence, given that the foreign-born population in the study area consisted mostly of economic immigrants from low-income countries, a high percentage of the excluded emergency contacts were probably made by tourists, a population beyond the scope of this study. We compared the age, sex, and specialty distribution among the emergency visits made by persons with and without health card, and no statistically significant differences were found when the whole group was studied or when the Spanish-born and the foreign-born population were studied separately.

The country of origin of the foreign-born residents studied, which, like the general distribution of immigrants in Spain, was diverse, does not limit the external validity of the results within Spain, although this validity is probably restricted to metropolitan areas. Nor is the study's external validity limited by the hospital's profile, a university tertiary hospital, since most hospitals and all the tertiary referral hospitals in Spain are public and people are assigned to a hospital (the closest to their homes), with no distinctions in quality or the people attended. However, the socioeconomic position and health status in the area of maximum attraction are lower than the average for the city of Barcelona. The *synthetic index of social inequalities *(which is constructed by taking into account life expectancy, the unemployment rate, the population rate with higher education, the population rate with insufficient education, and by comparing these values with the ideal rates) in 2001 of Ciutat Vella was 729, the lowest of all Barcelona's districts [35]. The Catalonian Health Survey [36] as well as other studies [14,27-29,32,37,38] have repeatedly shown that health services utilisation is inversely related to socioeconomic position. At least two aspects are related to this phenomenon: firstly, higher socioeconomic position is associated with healthier lifestyles and thus better health and lesser health services need and, secondly, adults with above-average incomes are more likely to have private supplemental insurance (double coverage, public and private) and thus tend to use public services less. These considerations could mean that, if determined in other settings with a Spanish-born population with better health and social and economic indicators, the differences in utilisation rates between Spanish-born and foreign-born residents would probably be smaller.

Although most of the population in Spain uses the national health service, there is a small percentage of the population that can choose the alternative of private healthcare, when available, to overcome national health service quality constraints (comfort aspects) and access barriers (waiting time and waiting lists for elective surgery.) In our study, the private sector did not play an important role for several reasons. Firstly, the private sector does not usually offer emergency hospital services. Secondly, in the study area, there are no private healthcare facilities and therefore people living in this area would have to travel to another area of the city to get private healthcare. Finally, if we had included a substantial group of people with double coverage and if double coverage had had an effect on public emergency department utilisation, these people would most probably have belonged to the Spanish-born group and therefore the real differences of frequency rates between the Spanish and the foreign-born population would have been underestimated.

Among the limitations of this study, some relate to the lack of some adjusting variables. The emergency department registry does not include information on socioeconomic status (SES), which has been shown in the literature to be related to differences in healthcare access [14,27-29,32,37,38]. The SES in the area of maximum attraction is low for both the Spanish-born and the foreign-born population, and we believe that, even if adjustment were made for SES, the differences would remain. Additionally, there is some evidence that SES plays less of a role in explaining differences in hospitalisation among immigrants than among native-born persons [20,21,27]. We believe that this is true in our area of low SES in general, although we are at present unable to prove this. Furthermore, because most immigrants arrived in Spain less than 5 years before the year of the study, we believe that healthy immigrant selection in the countries of origin and possible access barriers in the host country have a greater influence on their underutilisation of health services than their SES.

The lack of an individual indicator on health status in Spain prevented us from adjusting the utilisation rates by health. The healthy immigrant hypothesis used to explain their underutilisation, is based on studies that have measured health among recent immigrants to other countries [16,25] and some initial approaches in Spain [39]. In addition, the study's data sources do not include length of residence for immigrants in Barcelona or Spain and consequently adjustment by this variable, which also influences healthcare utilisation [16,31,40,41], could not be performed. Nevertheless, since less than 10% of the immigrants stay for more than 5 years in Barcelona, almost all foreign-born residents can be considered "recent" immigrants [3].

## Conclusion

Some useful conclusions for health policy can be drawn from the results of this study. Firstly, the lower utilisation of the emergency department by foreign-born residents compared with that among the Spanish-born population suggests that the population increase due to immigration does not translate directly into a corresponding increase in the number of emergency contacts in the area. Therefore, the impact of immigration on emergency departments is lower than would be expected if only the increase in the registered population was considered (and, as previously stated, is probably lower still on other healthcare services). Secondly, this latter conclusion does not contradict the finding of inappropriate use of the emergency department by foreign-born residents, since the differences in utilisation rates disappear in minor emergencies, which could probably be attended in primary care. This argument is supported by a previous study in our hospital showing that the cost of emergency visits by immigrants was lower than that of those made by the Spanish-born population, indicating that the immigrant population tends to access the health service through the emergency department even more than the Spanish-born population [9]. Moreover, other studies have shown no relationship between the perceptions of need, willingness to seek health services and ethnicity [42], and more evidence is provided towards the hypothesis that barriers occur at the access of the services [22,43]. Thus, the issue of barriers to access to other health services, especially among the foreign-born population, should be addressed. Access to healthcare information often leads to more appropriate utilisation of available resources. Finally, the greater utilisation of the gynaecological service of the emergency department by foreign-born women, as well as the higher complexity of these episodes [9], although not statistically significant, suggests that greater efforts and possibly direct interventions should be made to include women of reproductive age in antenatal care programs.

Future research could aim to confirm or refute our results in other healthcare settings, especially in primary health. In-depth study of barriers to other health services and other causes of the higher use of the emergency department among the foreign-born population is also required and could suggest ways to decrease barriers to access and promote more effective and efficient use of the different health services among the foreign-born and Spanish-born populations.

## Competing interests

The author(s) declare that they have no competing interests.

## Authors' contributions

All the authors have contributed to the achievement of this study. AB, FC and XC have made substantial contributions to conception and design, acquisition, analysis and interpretation of data, have been involved in drafting or revising the manuscript and have given final approval of the version to be published. OG and OV have participated in the acquisition and interpretation of data, and have been involved in revising the manuscript.

## Pre-publication history

The pre-publication history for this paper can be accessed here:


